# A Cadaveric Investigation of the Dorsal Scapular Nerve

**DOI:** 10.1155/2016/4106981

**Published:** 2016-08-15

**Authors:** Vuvi H. Nguyen, Hao (Howe) Liu, Armando Rosales, Rustin Reeves

**Affiliations:** ^1^Center for Anatomical Sciences, University of North Texas Health Science Center, Fort Worth, TX 76107, USA; ^2^Department of Physical Therapy, University of North Texas Health Science Center, Fort Worth, TX 76107, USA

## Abstract

Compression of the dorsal scapular nerve (DSN) is associated with pain in the upper extremity and back. Even though entrapment of the DSN within the middle scalene muscle is typically the primary cause of pain, it is still easily missed during diagnosis. The purpose of this study was to document the DSN's anatomy and measure the oblique course it takes with regard to the middle scalene muscle. From 20 embalmed adult cadavers, 23 DSNs were documented regarding the nerve's spinal root origin, anatomical route, and muscular innervations. A transverse plane through the laryngeal prominence was established to measure the distance of the DSN from this plane as it enters, crosses, and exits the middle scalene muscle. Approximately 70% of the DSNs originated from C5, with 74% piercing the middle scalene muscle. About 48% of the DSNs supplied the levator scapulae muscle only and 52% innervated both the levator scapulae and rhomboid muscles. The average distances from a transverse plane at the laryngeal prominence where the DSN entered, crossed, and exited the middle scalene muscle were 1.50 cm, 1.79 cm, and 2.08 cm, respectively. Our goal is to help improve clinicians' ability to locate the site of DSN entrapment so that appropriate management can be implemented.

## 1. Introduction

In standard anatomical textbooks and atlases, the dorsal scapular nerve (DSN) is documented as a motor nerve originating from the ventral ramus of spinal nerve root C5, from the superior trunk of the brachial plexus [1–4]. In addition to C5, various texts have also documented the DSN to occasionally receive contributions from C4 [5–9]. This nerve typically pierces the middle scalene muscle and travels posteroinferiorly to innervate the levator scapulae, rhomboid minor, and rhomboid major muscles [6–11]. Collectively, these muscles function to elevate and retract the scapula [4–6].

In contrast, several anatomical studies in the primary literature indicated that the spinal root origins and muscle innervations of the DSN may vary. One study found that the DSN not only receives contribution from C5 but also may receive variable contributions anywhere from C4-T1 [12]. Ballesteros' and Ramirez's study found that nearly 48% of the DSNs originated from C5 whereas approximately 30% shared a trunk with the long thoracic nerve [13]. Lee et al. (1992) reported that nearly 25% of the DSNs in their study originated from other spinal nerve roots aside from C5 [14] whereas Tubbs et al. (2005) reported that 95% of the DSNs originated from C5 and 5% branched from C5 and C6 [10]. A recent study by Shilal et al. (2015) also documented that the DSN arose from C5 and C6 and receives communications with the long thoracic nerve [15]. In addition, there are varying reports regarding the muscles that DSN innervates. For example, one case study from Japan reported that the DSN innervated the serratus posterior superior muscle [16]. In a study by Frank et al. (1997), they reported that the DSN innervated the levator scapulae muscle in only 11 out of 35 neck specimens [17].

The entrapment of the DSN is often located at the middle scalene muscle, because the nerve often pierces this muscle [18]. This nerve impingement or entrapment often leads to pain in the upper extremity and back. Patients typically experience sharp or aching pain along the medial border of their scapula that can radiate to the lateral aspect of their arm and forearm [19]. In addition, patients also report pain in their neck and back, as well as dysfunction of their shoulders [12]. Occupations which involve raising the arms over long periods of time, such as painters and electricians, make these particular individuals more likely to develop DSN entrapment [20]. There are also reports of DSN injury among athletes [21]. For example, Jerosch et al. (1990) reported that, along with injury to the long thoracic nerve, the DSN was also injured as a result of an anterior shoulder dislocation during judo [22]. Another report described an isolated DSN entrapment in a body builder using anabolic steroids. It was thought that the middle scalene muscle was injured due to repetitive stretching during exercises of neck flexion and forceful repetitive shoulder shrugging [23]. Lastly, concurrent with injury to the suprascapular nerve, the DSN was also injured in two sibling volleyball players. According to Ravindran, the brother and sister were active volleyball players for over 6 years and interestingly had almost identical symptoms in that both developed right shoulder and scapular pain with particular wasting of the right infraspinatus muscles. Both siblings also had mild winging of the right scapula with weakness of the rhomboid muscles [24]. In addition to these DSN injuries in sports, there are also case reports in which a lesion to or neuropathy of the DSN caused scapular winging [20, 25]. Because the DSN branches from the brachial plexus, clinicians often describe the impingement of this nerve as contributing to thoracic outlet syndrome (TOS). Specifically, the impingement of the DSN affects the interscalene space in TOS [12, 26, 27]. Meaningful epidemiological figures of this syndrome are difficult to obtain due to debate among clinicians with regard to the exact definition, diagnosis, and treatment of TOS [28]. As a result, some experts believe that TOS may be underdiagnosed or misdiagnosed [29–31]. The incidence of TOS has been broadly estimated to range from 0.3% to 8% in the US population [28, 32] and the most commonly affected age range is between 20 and 50 years [30].

Current treatments used by clinicians in relieving patients from DSN entrapment may involve either conservative and/or surgical treatments. Conservative treatments beyond physical rehabilitation may involve administering a local nerve block injection, which is commonly guided via ultrasound, in order to relieve patients of their symptoms [27, 33, 34]. It is very important for health care providers to have good working knowledge of the area around the scalene muscles in the neck, especially if they are going to apply nerve block injections in this area. They must be aware of other important neurological structures such as the roots and trunks of the brachial plexus and the phrenic nerve. Surgical treatment for DSN entrapment typically involves lesion of the muscle that is impinging the DSN, most often the middle scalene muscle [12]. In either case, it is important for clinicians to be mindful of the location and route of this nerve as it passes anterior, through, or posterior to the middle scalene muscle.

The purpose of this study is to undertake a more extensive investigation and description of the anatomy of the DSN in order to gain a better understanding of the spinal root origins, anatomical route, and muscular innervations of this nerve. In addition, we created a model of the DSN's path in relation to the middle scalene muscle by using measurements established at the transverse plane of the laryngeal prominence. The measurements for the DSNs in this study will assist clinicians with efficiency in pinpointing the surface location of this nerve in their patients for the purpose of diagnosis and treatment of possible nerve entrapment.

## 2. Materials and Methods

The dorsal scapular nerve was dissected and examined in 20 embalmed adult cadavers (12 females and 8 males) obtained through the Willed Body Program, Center for Anatomical Sciences, at the University of North Texas Health Science Center (UNTHSC) in Fort Worth, Texas. The age of the donors span from 52 to 93 years with a mean age of 75 years. The self-reported ethnicities of the donors are (95%) Caucasian and (5%) African American. The cadavers are individually wrapped in cotton shrouds with Maryland State Wetting agent (Hydrol Chemical Company, Yeadon, PA) and are stored in metal tanks located in the UNTHSC Gross Anatomy Laboratory.

The cadavers used in this project were initially dissected by first-year medical students enrolled in the school's gross anatomy course. Once the medical students were finished with their dissections, the final preparation of the cadavers for this study began. The sternocleidomastoid muscles were detached from their origin and reflected laterally. The superior trunk of the brachial plexus (C5 and C6) was identified between the anterior and middle scalene muscles and any fascia overlying these muscles was removed. The DSN was first identified in relation to the scalene muscles, and then the route to the muscles that it innervates was traced. If the DSN or the scalene muscles on the cadaver were damaged (left or right side), then the DSN data on that side was excluded from the study. The majority of the DSN dissection in this study remained intact on the left side of the neck region compared to the right one. On the right side of the neck, an incision was made to access vasculature for the embalming of our cadavers. Therefore, important structures such as the scalene muscles and the DSN were often damaged on that side.

**Figure 1:**
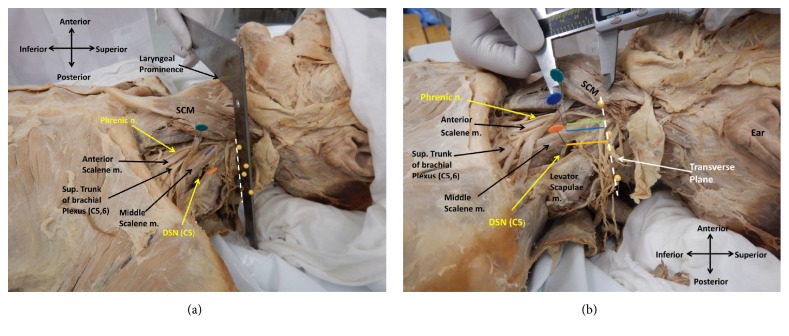
(a) A 90° angle ruler was placed directly on top of the laryngeal prominence creating a transverse plane as denoted by the yellow pins. The DSN branches from C5 and pierces the middle scalene muscle.** (**b**)** An electronic sliding caliper was used to measure the distances (cm) from the transverse plane (white dashed line) of the laryngeal prominence to the DSN as it enters the middle scalene muscle (green pin), crosses this muscle (blue pin), and exits the middle scalene muscle (orange pin). SCM is sternocleidomastoid muscle.

A transverse plane through the laryngeal prominence was established using a 90° angle ruler to create a reference site for three points of measurement to document the oblique route the DSN takes in relation to the middle scalene muscle. The points were derived measuring the distance of the DSN from this transverse plane as the nerve enters, crosses, and exits the middle scalene muscle. The point at which the DSN “enters” the middle scalene muscle is defined as where the nerve initially contacts the medial border of the middle scalene muscle. The point at which the DSN “exits” the middle scalene muscle is defined as where the nerve contacts the lateral border of the middle scalene muscle. Finally, the point where the DSN “crosses” the middle scalene muscle is defined as the midpoint where the nerve contacts the medial (enters) and lateral (exits) border of the muscle. Yellow pins were placed to delineate the transverse plane (white dashed lines) of the laryngeal prominence (Figure 1(a)). The distances of the DSN from this plane as it enters, crosses, and exits the middle scalene muscle were measured using an electronic sliding caliper (Mitutoyo Corp.); three repeated measurements were made for each observation from this plane (Figure 1(b)). Average values and standard deviations were calculated from these measurements. In order to test the reliability (consistency) of these measurements, Cronbach's alpha test was conducted through the Statistical Package for Social Sciences (SPSS) software (IBM Corp. 2015. IBM SPSS Statistics for Windows, Version 23.0, Armonk, NY: IBM Corp.). Dissection images were taken with a digital camera (Nikon Coolpix AW110).

## 3. Results

**Figure 2:**
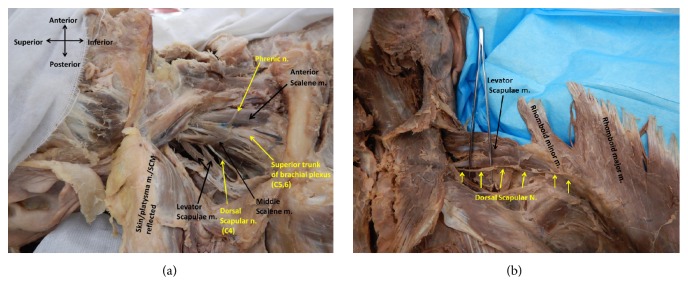
(a) An anterolateral view of the right neck region of a 90-year-old female cadaver in the supine position. The DSN branches from C4 and pierces the middle scalene muscle. (b) In the prone position, the DSN travels posteroinferiorly after piercing the middle scalene muscle to supply the levator scapulae, rhomboid minor, and rhomboid major muscles. The rhomboid muscles are reflected laterally from their origin to show the route of the DSN.

The DSN was dissected from 20 embalmed adult cadavers that were previously dissected by first-year medical students for classroom study. From these 20 cadavers, a total of 23 DSNs were examined. As indicated in Table 1, 70% of the DSNs originated from the spinal nerve roots of C5, whereas 22% arose from C4 and 8% from C6. With regard to the route of the DSN in relation to the middle scalene muscle, 74% pierced the muscle, whereas 13% of the DSNs traveled anterior to the middle scalene muscle, and 13% traveled posterior to the muscle. In addition, we observed that 52% of the DSNs provided innervation to the levator scapulae, rhomboid minor, and rhomboid major muscles combined. Furthermore, we observed that, in 48% of the cadavers in our study, the DSN supplied only the levator scapulae muscle. Figures 2(a) and 2(b) are examples of a dissection of the DSN followed from its spinal root origins, anatomical route in relation to the middle scalene muscle, and muscular innervations in a 90-year-old female cadaver in the supine and prone position, respectively.

Measurements were taken of the DSN as it courses obliquely from the medial to lateral border of the middle scalene muscles. From the transverse plane of the laryngeal prominence, the mean distance at which the DSN enters (medial border) the middle scalene muscle was 1.50 cm with a standard deviation of 0.88 cm; the DSN crosses (midpoint) the middle scalene muscle at 1.79 cm (±0.89 cm) and exits (lateral border) this muscle at a mean distance of 2.08 cm (±0.96 cm). These mean values and standard deviations were calculated from the 23 DSNs dissected and documented in this study (*N* = 23). Cronbach's alpha value was 0.999 which indicates very high consistency of the triplicate measurements conducted in this study.

## 4. Discussion

In this study, we report the percentage of cases in which the spinal root of the DSN arose from C5 (70%) to be very similar to that reported by Lee et al. (1992) where the DSN arose from C5 approximately 75.8%. Similar to our data, almost 25% of the DSNs in that study originated from spinal root origins other than C5, such as from the superior trunk of the brachial plexus (C5 and C6), C4 and C5, and C6 alone [14]. In terms of the muscular innervations, almost half of the DSNs in our study supplied the levator scapulae muscles only. Interestingly, Frank et al. (1997) reported that although the DSN consistently pierced the middle scalene muscle, the muscular innervations of this nerve were highly variable. Their study documented that the DSN innervated the levator scapulae in only 11 out of 35 neck specimens [17]. These reports and our current data suggest that the anatomy of the DSN is variable and may be a possible reason in which clinicians often overlook the impingement of this nerve during differential back diagnosis [18].

## 5. Conclusion

Our research will assist clinicians in becoming aware of potential variations in the overall anatomy of the DSN in terms of its spinal root origins, anatomical route, and muscular innervations. No prior study has measured the oblique route of the DSN as it crosses the middle scalene muscle relative to the transverse plane of the laryngeal prominence. For future studies, these measurements will allow us to evaluate the surface projection of the DSN relative to its typical site of impingement (the middle scalene muscle) while using the transverse plane of the laryngeal prominence as a reference point. The long-term goal of this study is to provide data to assist clinicians and therapists to accurately and efficiently pinpoint the location of this nerve in patients with possible DSN impingement.

## Figures and Tables

**Table 1:** Variation in the spinal roots and innervations of the DSN.

	*N* and percentage
*Origin*	
C4	5 (22%)
C5	16 (70%)
C6	2 (8%)

*Route*	
Anterior to middle scalene m.	3 (13%)
Piercing middle scalene m.	17 (74%)
Posterior to middle scalene m.	3 (13%)

*Muscles innervated*	
Levator scapulae m. only	11 (48%)
Levator scapulae m. & rhomboid mm.	12 (52%)

*Total N*	*23*

Cadaver number (*N*) and percentage for specific spinal root origins, route, and muscles innervated for the DSN.
